# The complete mitochondrial genome sequence of the green microalga *Lobosphaera* (*Parietochloris*) *incisa* reveals a new type of palindromic repetitive repeat

**DOI:** 10.1186/s12864-015-1792-x

**Published:** 2015-08-05

**Authors:** Nicolas J. Tourasse, Nastassia Shtaida, Inna Khozin-Goldberg, Sammy Boussiba, Olivier Vallon

**Affiliations:** Institut de Biologie Physico-Chimique, UMR CNRS 7141 - Université Pierre et Marie Curie, Paris, France; Institut de Biologie Physico-Chimique, FRC CNRS 550, Université Pierre et Marie Curie, Paris, France; Microalgal Biotechnology Laboratory, French Associates Institute for Agriculture and Biotechnology of Drylands, J. Blaustein Institutes for Desert Research, Ben-Gurion University of the Negev, Midreshet Ben-Gurion, 84990 Israel; ARNA Laboratory, INSERM UMR 869, Université Bordeaux 2, Bordeaux, France

**Keywords:** Chlorophyta, Trebouxiophyceae, *Myrmecia*, Replication origin, Genome rearrangement, LIMP, HyLIMP, DNA cruciform, Palindromic repeat, Transcript processing

## Abstract

**Background:**

*Lobosphaera incisa*, formerly known as *Myrmecia incisa* and then *Parietochloris incisa*, is an oleaginous unicellular green alga belonging to the class Trebouxiophyceae (Chlorophyta). It is the richest known plant source of arachidonic acid, an ω-6 poly-unsaturated fatty acid valued by the pharmaceutical and baby-food industries. It is therefore an organism of high biotechnological interest, and we recently reported the sequence of its chloroplast genome.

**Results:**

We now report the complete sequence of the mitochondrial genome of *L. incisa* from high-throughput Illumina short-read sequencing. The circular chromosome of 69,997 bp is predicted to encode a total of 64 genes, some harboring specific self-splicing group I and group II introns. Overall, the gene content is highly similar to that of the mitochondrial genomes of other Trebouxiophyceae, with 34 protein-coding, 3 rRNA, and 27 tRNA genes. Genes are distributed in two clusters located on different DNA strands, a bipartite arrangement that suggests expression from two divergent promoters yielding polycistronic primary transcripts. The *L. incisa* mitochondrial genome contains families of intergenic dispersed DNA repeat sequences that are not shared with other known mitochondrial genomes of Trebouxiophyceae. The most peculiar feature of the genome is a repetitive palindromic repeat, the LIMP (*L. Incisa* Mitochondrial Palindrome), found 19 times in the genome. It is formed by repetitions of an AACCA pentanucleotide, followed by an invariant 7-nt loop and a complementary repeat of the TGGTT motif. Analysis of the genome sequencing reads indicates that the LIMP can be a substrate for large-scale genomic rearrangements. We speculate that LIMPs can act as origins of replication. Deep sequencing of the *L. incisa* transcriptome also suggests that the LIMPs with long stems are sites of transcript processing. The genome also contains five copies of a related palindromic repeat, the HyLIMP, with a 10-nt motif related to that of the LIMP.

**Conclusions:**

The mitochondrial genome of *L. incisa* encodes a unique type of repetitive palindromic repeat sequence, the LIMP, which can mediate genome rearrangements and play a role in mitochondrial gene expression. Experimental studies are needed to confirm and further characterize the functional role(s) of the LIMP.

## Background

*Lobosphaera incisa* (Reisigl) comb. nov. is a unicellular green alga belonging to the class Trebouxiophyceae (phylum Chlorophyta), which includes coccoid or pseudo-filamentous species from subaerial, soil, or freshwater habitats, and lichen photobionts. *L. incisa* was originally assigned to the genus *Myrmecia*, but subsequently reclassified as *Parietochloris* and then as *Lobosphaera* [1–3]. Based on chloroplast genome sequences, *L. incisa* was recently placed in clade C, the most derived of the Core Trebouxiophyceae [4]. *L. incisa* is an oleaginous alga and a target organism of high biotechnological interest because it is the richest known plant source of arachidonic acid, a pharmaceutically and nutraceutically valuable ω-6 long-chain polyunsaturated fatty acid that accumulates in considerable amounts when cells are cultivated under specific conditions such as nitrogen starvation [5, 6]. Recently, a nuclear transformation system has been developed for *L. incisa* [7] and the complete sequence of its chloroplast genome has been released [8]. Here we report the complete sequence of the mitochondrial genome of *L. incisa* determined from high-throughput Illumina short-read sequencing. We compare it with the nine other mitochondrial genomes reported for Trebouxiophyceae. We focus on the analysis of the main feature of the genome, a novel type of palindromic repeat sequence.

## Results

### *Feature content and organization of the* L. incisa *mitochondrial genome*

**Fig. 1:**
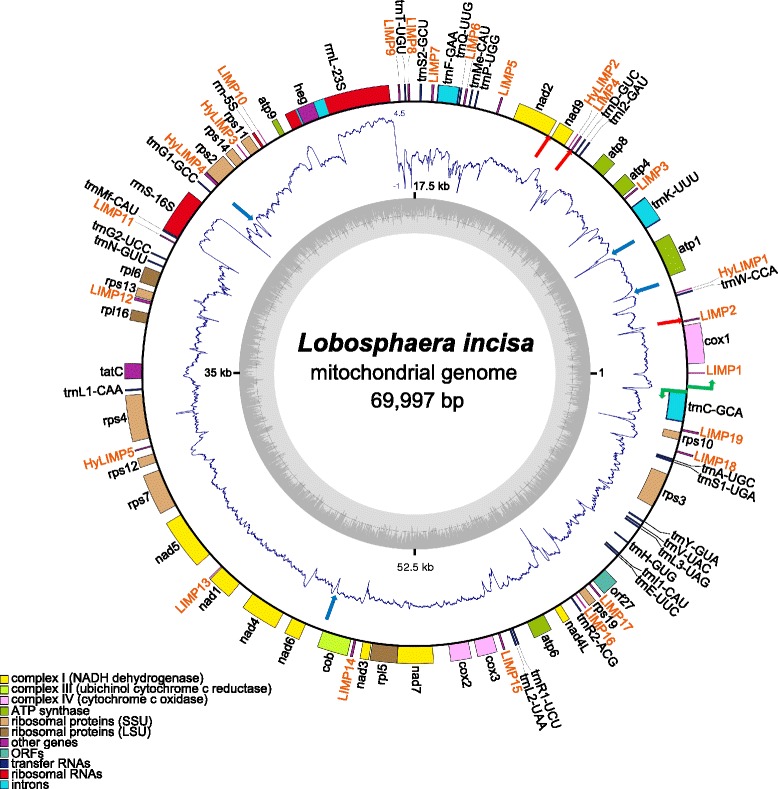
Schematic representation of the *L. incisa* mitochondrial genome. Genes depicted on the outside of the outer circle are transcribed in counterclockwise orientation, on the inside in clockwise orientation. Putative promoters are shown as green bent arrows. Genes are color-coded according to functional classes, as listed in the bottom left corner. The LIMP and HyLIMP copies are shown in orange. The inner ring displays a graph of the G + C content (the circle marks the 50 % threshold), the middle one the RNA-Seq coverage (log_10_ scale, with 0 values brought to −1). Red arrows indicate the locations of the three short regions that are deleted in a fraction of the molecules, and blue arrows indicate the locations of the four palindromes (not LIMPs) of high predicted stability that are associated with a drop or loss of RNA-Seq coverage. The figure was generated using OrganellarGenomeDRAW [74]. The coverage plot was drawn using the “polar.plot” function from the plotrix package in R

Starting with scaffolds and contigs built from paired-end (PE) and mate-pair (MP) sequencing libraries using several assembly programs run with various parameter settings, the assembly of the mitochondrial genome of *L. incisa* was manually closed into a single, continuous, circular-mapping sequence of 69,997 bp (Fig. 1). The assembly presented here is based on ~5000X sequencing coverage (3,889,871 mapped reads) and therefore represents a high-quality and high-confidence sequence. It is the most massively consistent with the high-throughput sequencing reads, but mapping of the reads revealed three loci, all intergenic, with noticeable heterogeneity. They correspond to short deletions (173, 110 and 57 nt; red arrows in Fig. 1) in a fraction of the molecules, due to short direct repeats (respectively 9, 12 and 11 nt long). In addition, minor changes in the length of short poly-A or poly-T tracts were observed at 68 positions.

The *L. incisa* mitochondrial genome exhibits a G + C content of ~36 %, and is predicted to encode 64 genes: 34 protein-coding genes (total coding capacity, 42 %), 3 rRNAs, and 27 tRNAs (Fig. 1), a gene repertoire similar to that found in the nine known Trebouxiophyceae mitochondrial genomes [9–15]. tRNAs are present for all amino acids and three of them are interrupted by group II introns (*trnC*-GCA, *trnF*-GAA and *trnK*-UUU). A self-splicing group I intron, possibly mobile as it encodes a LAGLIDADG homing endonuclease, is inserted in the 23S rRNA gene. The noncoding part of the intron shows similarity to introns found in the mitochondrial *rrnL* genes of *Chlorella variabilis* and *Trebouxia aggregata*. But its homing endonuclease belongs to subfamily 2 and rather resembles similar enzymes encoded in chloroplast *rrnL* introns of other Trebouxiophyceae, which suggests inter-organelle intron trafficking.

In the mitochondrial genome of *L. incisa*, the genes are distributed in two clusters read on opposite DNA strands, comprising 53 and 11 genes, respectively (Fig. 1). From this bipartite organization, one can presume that the genome is expressed in two transcriptional units produced from divergent promoters, as found in animal mitochondria and experimentally demonstrated in the Trebouxiophyceae *Prototheca wickerhamii* [16]. Between *cox1* and *trnC-GCA*, we found two divergent motifs (TATATAGAA and TTTATAGGA at positions 69264 and 69295, on the minus and plus strands, respectively) resembling one of the *P. wickerhamii* promoter sequences TATATAG**G**A (where the first nt of the transcript is underlined). In support of this assignment, RNA-Seq transcriptome coverage is almost nil between these positions and increases a few nucleotide downstream.

The *L. incisa* mitochondrial genome harbors seven families of various dispersed DNA repeat sequences, totaling 66 copies for 3.9 kb, i.e. 5.6 % of the genome size (Table 1). Repeats are defined here as sequences ≥30 nt in length found more than 4 times on the genome. All are located in intergenic regions. The average G + C content of the repeat families is similar to that of the whole mitochondrial genome (34–45 %). The dispersed repeat sequences are completely different from the repeats identified in the chloroplast genome of *L. incisa* [8] and are virtually absent from the *L. incisa* nuclear genome (at most two isolated hits for a given element, unpublished data). Comparison with the other mitochondrial genomes of Trebouxiophyceae reveals that all seven repeat families are unique to *L. incisa*. In addition, an array of 12 repetitions of the nonanucleotide GAGGGCTAC, also not found in other species, is located downstream of *rps12*.

**Table 1:** Families of interspersed DNA repeats identified in the *L. incisa* mitochondrial genome

Repeat name	# of copies	Consensus sequence^a^	Notes
LIMP	19	(AACCA)_*m*_[AATGAAA or TTTCATT](TGGTT)_*n*_	Repetitive palindrome; 2 ≤ (*m*,*n*) ≤13
Repeat_2	16	GCCTGTACAAATCTCTGCCCAACCGTAATGAAATGGTTGGCAAAAGAAAAAGAAATGGTGAGAGTAATCAAATGGTTGGCTC	Three copies interrupted by a LIMP; four copies next to LIMPs
Repeat_3	6	CCAGTTAAAATGAATGGCAAAAAACAAATGGTTGG	
Repeat_4	8	CTCCACACCATTTCATTAATCTCTGATTTGTTCA	
Repeat_5	7	TTTGGTTTGGTTTGGTTACAAATCAGAGAAAAGCAGGGGCTC	Six copies overlapping LIMPs
Repeat_6	5	TTACGGTTGGGCAGAGAAAAAAGGCAACCGTGAAAAAAAAAGCTGCGGT	three copies overlapping LIMPs
Repeat_7	5	AATCTCTGATTTGTTCAGGAACAACTGGTTGGG	All copies adjacent to LIMPs

^**a**^palindromic positions are underlined

### A novel palindromic repeat, the LIMP

**Fig. 2:**
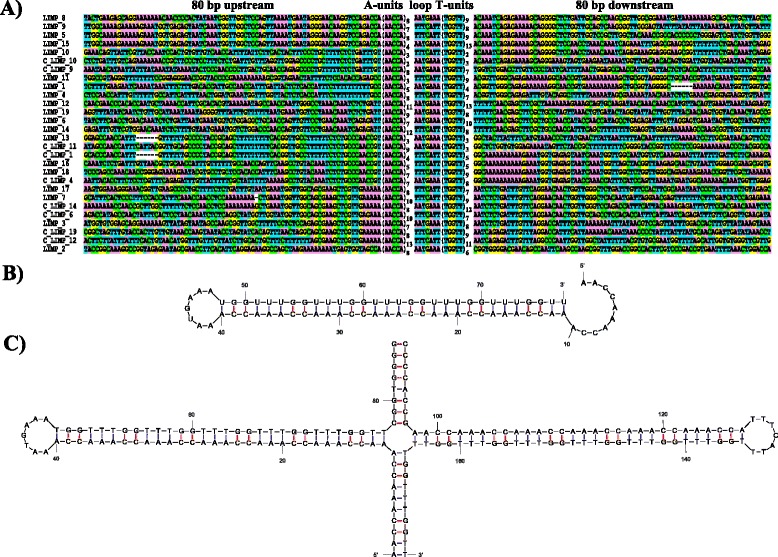
Sequence and predicted secondary structure of LIMP repeats. **a** Alignment of all LIMP copies along with 80 bp of flanking sequence from both sides. LIMPs have been ordered so as to see the similarity between their flanks. For nine LIMPs, the reverse-complemented sequence is also shown to illustrate the fact that a few LIMPs have similar complementary flanks. **b** Predicted single-stranded RNA secondary structure of a LIMP, using LIMP #2 as an example. **c** Predicted double-stranded DNA secondary structure of the cruciform at LIMP #2

One of the repeats in the mitochondrial genome of *L. incisa* stands out as highly unusual, because it is both palindromic and internally repetitive. We termed it the LIMP (*L. IncisaM*itochondrial *P*alindrome). Nineteen copies are found in the genome (Table 2). Their structure can be summarized by the following pattern: 5’-(AACCA)_*m*_[AATGAAA or TTTCATT] (TGGTT)_*n*_-3’, where 2 ≤ (*m*,*n*) ≤13 (Table 2 and Fig. 2a). Each LIMP is by itself repetitive, as it comprises a pentanucleotide motif (AACCA) that is repeated a variable number of times, and palindromic as the complementary motif TGGTT is repeated downstream, after a short loop. Hereafter, AACCA and TGGTT pentanucleotides will be referred to as “A-units” and “T-units”, respectively. The number of A- and T-units for a given LIMP can differ. The two branches of the stem are separated by a conserved 7-nt loop that can be found in either of two orientations: AATGAAA or TTTCATT (reading on the plus strand). The first orientation will hereafter be referred to as +, the second one as -. LIMP copies are always intergenic, and found in both the clockwise and the counterclockwise gene clusters, with + and - orientations about equally represented (Table 2, Fig.2a). On the RNA, the LIMP sequence will always read 5’-AACCA…UGGUU-3’, while the loop will read either AAUGAAA or UUUCAUU. While the loop sequence is invariant, variants of the pentanucleotide can be found just next to some LIMPs (A*G*CCA and *C*GGT*G* flanking LIMP #2; *GT*CCA *G*ACCA left of LIMPs #5, 8, 9 and 15, which leads to an increase in the length of the palindrome and in some cases makes the LIMP almost symmetrical; see Fig. 2a). As another evidence for mutational decay of the LIMP ends, 79 LIMP remnants were also found in the genome, containing a perfect or near-perfect 11–20 nt fragment of the LIMP (including the loop) but unable to form a stem-loop. These remnants often reside near *bona fide* LIMPs or are associated with other repeat sequences.

**Table 2:** Features of LIMP repeats identified in the *L. incisa* mitochondrial genome

LIMP #	Center^a^	Structure^b^	Total length (nt)	Stem length (bp)	Palindromic region^c^(nt)	Imperfect genomic reads^d^	ΔG RNA hairpin (kcal.mol^−1^)^e^	transcriptome coverage^f^
1	33	5;+;4	52	20	52	0;6	−30.60	low (19)
2	2152	8;+;6	77	30	77	18;5	−49.70	NO (2)
3	7742	7;+;8	82	35	82	0;11	−63.00	NO (1)
4	10697	8;-;7	82	35	82	0;6	−61.20	NO (0)
5	14093	8;-;9	92	40	96	9;7	−77.60	NO (1)
6	15481	7;+;10	92	35	92	1;2	−66.60	NO (1)
7	16799	10;-;9	102	45	102	4;12	−80.00	NO (1)
8	17732	8;+;9	92	40	96	20;5	−76.80	NO (0)
9	18115	7;-;8	82	35	86	12;0	−68.20	NO (0)
10	23885	3;-;2	32	10	74	1;0	−55.20	normal (145)
11	29424	3;-;9	67	15	67	1;6	−27.40	normal (47)
12	32075	11;+;13	127	55	127	27;12	−101.70	NO (1)
13	43731	3;+;3	37	15	37	4;0	−21.70	normal (83)
14	50059	12;-;8	107	40	107	1;7	−70.40	NO (5)
15	55976	4;+;13	92	20	96	8;10	−48.00	low (13)
16	59523	5;-;5	57	25	57	0;0	−41.30	low (18)
17	60233	8;-;7	82	35	82	0;1	−61.20	NO (1)
18	66788	7;-;9	87	35	87	4;9	−63.10	NO (1)
19	67694	9;+;8	92	40	92	14;6	−68.20	NO (1)
average:			80.7	31.8	83.7		−59.6	
total:			1533	605	1591	124;105		

^a^position of first nt of first T-unit

^b^number of A-units; type of loop; number of T-units

^c^including additional nucleotides extending the stem, see Fig. 2a

^d^reads showing a change in the number of A-units; of T-units

^e^including extension of palindrome for #5, 8, 9 and 10

^f^coverage level (number of reads at position of lowest coverage; 5 or below is considered an interruption)

A striking feature of the LIMPs is the variable number of A- and T-units, which make the LIMP sequences vary from 32 to 127 bp in length and form asymmetric palindromes in 17 of the 19 cases (Table 2). In search for evidence of variability in the number of repeats, we extracted the genomic reads mapping to the LIMPs in an imperfect manner (i.e., with indels or with soft-clipping at one of the ends). When examined using the genome browser IGV [17, 18], only 229 reads were found to unambiguously indicate a number of repeat units different from that in the reference genome assembly (Table 2). Compared to the 62,478 reads that mapped to the LIMPs, this indicates a very low degree of variability within the population of DNA molecules sequenced. In particular, this analysis confirms that most LIMP stems in the genome are indeed asymmetrical, i.e., with different numbers of A- and T-units.

The propensity of the LIMPs to form hairpins as RNA and cruciforms as DNA, as depicted on Fig. 2b and c, respectively, was evaluated using mfold [19], a program that calculates the free energy (ΔG) of a DNA or RNA secondary structure. For the RNA hairpin, ΔG ranged between −101.7 and −21.7 kcal/mol (Table 2), compatible with the formation of at least some of these structures *in vivo*. To evaluate the propensity of the DNA to form cruciforms at the LIMP loci, we compared the free energy of the linear double-stranded B-DNA with that of the second-best structure, a cruciform where each strand folds onto itself according to the palindromic nature of the LIMP. As expected, the cruciform structure was markedly less stable, with an average difference 23.3 +/− 0.2 kcal/mol. This indicates that a DNA cruciform can form only if negative super-helicity is imposed to the molecule (see Discussion).

### LIMPs can be sites of genome rearrangements

Remarkably, all LIMPs share (virtually) identical flanking sequences with at least one other LIMP (Fig. 2a). The regions of identity are short, covering between 15 and 70 bp immediately next to the LIMPs, except for LIMPs #5 and 15 that share 104 bp of left flank and for LIMPs #10 and 15 that share 114 bp of right flank. Some LIMPs share only one flank (e.g., LIMPs #1 and 11), others show identity on both sides (e.g., LIMPs #16 and 18), and there are LIMPs sharing reverse-complemented flanks, where the left flank of a LIMP corresponds to the complementary sequence of the right flank of another LIMP or vice-versa (e.g., LIMPs #11 and 13 or LIMPs #7 and 14). For LIMP #10, the shortest of all LIMPs, the right flank is the reverse-complement of the left flank, so that the palindrome in this case is extended by 21 bp on each side. In a couple of instances the identical flanks actually correspond to copies of intergenic dispersed sequences that are found at other positions on the genome (Table 1). For example, LIMPs #4, 16 and 18 (Fig. 2a) are inserted within copies of repeat_2 that is found 16 times in the genome.

**Fig. 3:**
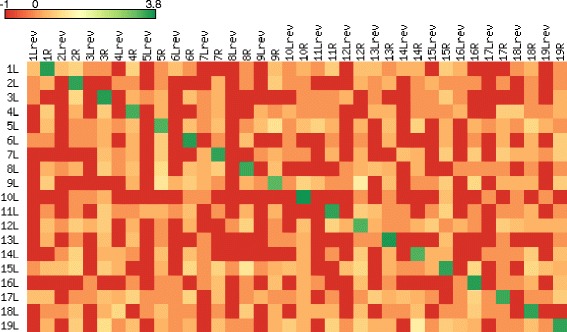
Heat map of interLIMP joints, generated using matrix2png 1.2.2 [75]. The map gives a colored representation of the number of read pairs that could connect all possible combinations of flanks between the 19 LIMPs (L, left flank; Lrev, reverse-complemented left flank; R, right flank). Each cell in the map represents the log_10_-transformed number of read pairs for which one mate was located on each side of the LIMP flank combination considered. If there was no pair, the value was set to −1 (red)

**Fig. 4:**
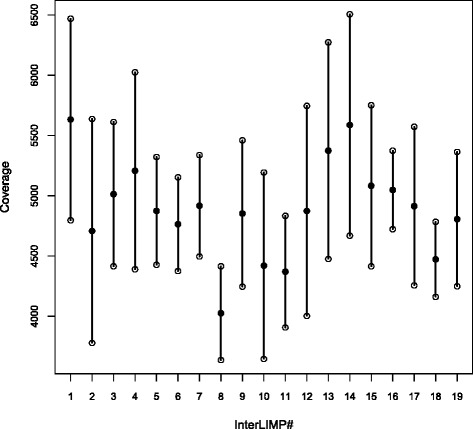
Single read coverage of interLIMP regions. For each interLIMP, the filled dot indicates the average coverage, and the lower and upper open dots represent the values of average ± standard deviation. InterLIMP #*i* corresponds to the region in-between LIMPs #*i* and *i + 1*, and interLIMP #19 is the region between LIMPs #19 and 1 (see Table 2)

This similarity among LIMP flanks most likely reflects the history of their duplication. At the same time, it predictably increases the probability of recombination between LIMPs. Indeed, one of the minor variants observed in our genomic reads (see above) was a deletion of LIMP #4, due to recombination between its last T-units and a short LIMP remnant found 110 nt upstream. To further test the hypothesis that LIMPs can be sites of recombination, we mined the genomic sequencing reads for the occurrence of DNA fragments connecting the flanks of different LIMPs. To this end, we counted the number of fragments uniquely mapping to the 722 possible combinations of left and right flanks from the 19 LIMPs (using 400 bp of flanking sequences or up to the next LIMP; Fig. 3). Read pairs connecting the flanks of LIMPs that are distant in our assembly were extremely rare, at most 14, compared to 2,100-6,300 for the original LIMPs (cells on the diagonal). This suggests that LIMPs can indeed mediate mitochondrial genome rearrangements, but that the vast majority of the molecules in our culture conform to our genome map. Examination of total read coverage shows some variability among interLIMPs (i.e., regions in-between two LIMPs; Fig. 4), suggesting copy-number variation for interLIMPs. But because there is no correlation with the rare recombination events described above, we propose that this copy number variation is due to a bias in replication (see Discussion) and not to recombination between LIMPs.

### LIMPs correspond to sites of low RNA-Seq coverage

Because of their palindromic nature, LIMPs have the potential to form stable stem-loop structures when transcribed as an RNA (see Table 2 and Fig. 2b). This raises the question of whether they could exert a function in transcript processing, i.e. help process the putative primary transcripts generated from the divergent promoters into shorter RNAs. We mapped the *L. incisa* paired-end and single-end transcriptomic reads to the mitochondrial genome assembly and found an average coverage of ~760X (~130X if the highly represented rRNAs are excluded). This indicates that, owing to their A/T-richness, the mitochondrial rRNAs and mRNAs were efficiently retained during poly-A RNA purification, the first step of the RNA-Seq protocol. Overall, coverage by RNA-Seq reads was relatively constant, with only a few regions showing an interruption of coverage. It therefore appears that many of the RNA molecules still comprise several cistrons, i.e. that maturation of the two precursor transcripts derived from the divergent promoters is not very efficient. Owing to their size, the tRNAs cannot be sequenced by our RNA-Seq method, yet all tRNA loci showed normal or slightly diminished coverage, indicating that they were only partially excised from the precursor transcripts. While interruption of coverage was sometimes seen near the 3’ end of certain tRNAs, it is clear that the “tRNA punctuation” mode of transcript processing described in mitochondria of animals and to some extent yeast and red algae, relying on precise excision of the tRNAs from the primary transcript [20–22], is not predominant in the mitochondrion of *L. incisa*. The introns in tRNA genes and even that in *rrnL* were highly covered by RNA-Seq data, with many reads spanning the junctions, indicating that these introns are not spliced from the primary transcript but after excision of the tRNA and rRNA precursors.

Interestingly, LIMPs showed an overall lower coverage (~33X) compared to the whole mitochondrial genome (Fig. 1). In particular, we found that 13 of the LIMPs were associated with a complete or almost complete loss of RNA-Seq coverage (Table 2). Only LIMPs #10, 11 and 13 showed a coverage comparable to that of the surrounding regions, while LIMPs #1, 15 and 16 showed decreased but significant coverage. Interestingly, there was a strong correlation with the number of pairable A- and T-units, i.e. all LIMPs with stems shorter than 20 nt showed no coverage interruption, while those with stems longer than 25 nt showed complete interruption (Table 2). A partial effect was observed with stems of 20 or 25 nt. The short LIMP #10, in spite of the additional stability conferred by the extended palindrome, did not interrupt RNA-Seq coverage.

### Another family of repetitive palindromes, the HyLIMP

**Fig. 5:**
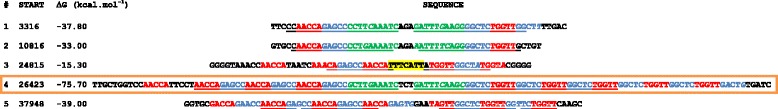
HyLIMP sequences. HyLIMP #4 which interrupts RNA-Seq coverage is boxed. Palindromes are underlined, and the reported ΔG value is for the RNA form of the palindromic sequence. In the sequences, the repeat units common with LIMPs are written in red, the other pentanucleotide in blue and the conserved inner part of the palindrome in green. Residues differing from the consensus are italicized. The region in HyLIMP #3 that is identical to the LIMP loop is highlighted in yellow

We systematically searched the mitochondrial genome for palindromes using the program Palindrome from the EMBOSS 6.4.0 package [23], requesting a stem longer than 15 bp and a loop shorter than 21 nt. Among the 27 palindromes found (excluding the LIMPs), four, those with the highest predicted stability (ΔG < −65 kcal.mol^−1^), were associated with a drop or loss of RNA-Seq coverage (blue arrows in Fig. 1). Three of them, unrelated to each other, were not internally repetitive and in two cases they obviously derive from the head-to-head assembly of two repeat elements (repeat_2 and repeat_4, respectively), probably brought together by recombination. But the fourth one (boxed sequence in Fig. 5) showed internal repetitivity in the stem, similar but different from that observed in the LIMPs. Its stem is made of three repeats of the 10-nt AACCA**GAGCC** sequence, i.e. a combination of the A-unit of a LIMP with a GAGCC pentanucleotide, prolonged by an unrelated decanucleotide. After a 3-nt loop, the complementary branch of the stem is found, with 5.5 repeats. This palindrome actually belongs to a small family of five related sequences (Fig. 5), all detected as palindromes by the criteria above. We call these repeats HyLIMPs, because of the hybrid nature of their repeat unit, partly similar to LIMPs. The other four HyLIMPs show the characteristic AACCAGAGCC-based palindrome, but they do not interrupt RNA-Seq coverage and their predicted stability is lower (−22 kcal.mol^−1^ > ΔG > −48 kcal.mol^−1^). Similar to LIMPs, HyLIMPs are often asymmetric, with one to three decanucleotide units in their stem. In HyLIMPs #1, 2 and 4, the non-repetitive inner part of the stem and the loop are conserved. Some sequence variability can be observed in the HyLIMP stem (italicized in Fig. 5), usually with complementary mutations in the other branch restoring Watson-Crick base-pairing. Like LIMPs, HyLIMPs show evidence for a dynamic evolution, with over 100 short sequences resembling HyLIMP #4 but not palindromic by our criteria, dispersed throughout the genome. As further proof of the relation between HyLIMPs and LIMPs, a HyLIMP decanucleotide-like sequence was found just left of LIMP #11 (see Fig. 2a), and the loop of HyLIMP #3 resembled that of the LIMPs (see Fig. 5). Fifty of these HyLIMP remnants were limited to the central part of the palindrome (in green in Fig. 5). Interestingly, this short palindrome is also found repeated 12 times in the chloroplast genome of *L. incisa* [8], and very similar repeats were found in the chloroplast genomes of three other Trebouxiophyceae of clade C, *Xylochloris irregularis*, *Leptosira terrestris* and *Dictyochloropsis reticulata*. No related sequence was found in the available Trebouxiophyceae mitochondrial genomes.

## Discussion

Based on its relatively high percentage of coding regions (42 %), large gene repertoire (64 genes) and limited extent of repeats (5.6 %), the *L. incisa* mitochondrial genome can be classified as belonging to the “ancestral” type of algal mitochondrial DNA, as opposed to the “reduced-derived” pattern observed in *Pedinomonas minor* and Chlorophyceae [10]. Overall, the gene content is highly similar to that in the nine available mitochondrial genomes of Trebouxiophyceae [9–15]. In contrast to this conserved gene repertoire, highly diverged gene order and orientation have been observed among Trebouxiophyceae [10–16] including the present species. Even though there are remaining blocks of synteny shared between the mitochondrial genomes of Trebouxiophyceae, this diversity in organization highlights the numerous genome rearrangements that have occurred since the group originated. It also implies that the mitochondrial genome is transcribed differently in the various species.

The most striking feature of the *L. incisa* mitochondrial genome is undoubtedly the presence of the LIMP. This repetitive palindromic repeat uses a fixed pentanucleotide ACCAA, repeated in direct and inverse orientation (A- and T-units) to form the two branches of a stem, with a fixed loop in-between. In the genome, LIMPs occur about equally in the two possible orientations, with no defined alternation pattern. No sequence similar to the LIMP can be found in any other organellar genome (chloroplast or mitochondrion) of Chlorophyta, and it therefore appears to have recently originated in the lineage leading to *L. incisa*. It is common for organellar genomes of algae to carry complex sets of short dispersed repeats (see e.g., [12, 24–31]), and each time they seem lineage- or even species-specific (in the case of *Volvox*). In some cases, repeat units can form extended tandem arrays [10, 32] or palindromes [26, 28, 33]. In *Volvox carteri*, both organelle genomes are bloated with repeats that form short (but non-repetitive) palindromes [26, 29]. Based on their large number and high sequence similarity, they have been described as selfish genetic elements, multiplying vigorously and spreading from the mitochondrial to the plastidial and even to the nuclear genome [26]. However, the stabilization of such structures in a polyploid genome where an efficient homologous recombination can rapidly correct the copy where the repeat would have duplicated, leads us to think that the repeats themselves confer a selective advantage to the cell, in other words that they have a function.

The two branches of the LIMP are rarely of equal length, implying that the stem formed by their annealing needs not be extremely long to perform its function (it will only be as long as the shortest of the branches). Presumably, the repetitive nature of the palindrome allows expansion of the stem via DNA polymerase slippage, as is observed in microsatellites [34–36]. Indeed, detailed analysis of imperfectly matching reads shows that insertions and deletions of repeat units can occur, observable even within the culture we have analyzed. This could counterbalance stem shortening via mutational decay, attested by the imperfect A- and T-units next to LIMPs #2, 5, 8, 9 and 15 (Fig. 2a). The absolutely invariant sequence of the loop is another indication that LIMPs have a function. What then could be this molecular function? Two categories of hypotheses can be envisioned: LIMPs may act at the DNA level, imposing a local cruciform structure to the chromosome, or at the RNA level, by forming a stem-loop.

Palindromic sequences in DNA have the potential to form cruciform structures, with each strand folded as a stem-loop over the palindromic region. Because the energy of the linear form is lower for a molecule with unconstrained ends (in the case of the LIMPs, by about 23 kcal/mol), cruciforms are believed to form only if negative supercoiling is imposed on the molecule [37]. A higher degree of supercoiling favors the “folded” conformation, where the two stems stack onto the linear part of the chromosome, a conformation that is required for such processes as recombination and transposition. A folded cruciform indeed resembles a Holiday junction [38], and its “resolution” can lead to a double-stranded break and recombination [39, 40]. Another possible mechanism leading to double-stranded break is the formation of a single-stranded hairpin on the lagging strand during DNA replication [39]. Whatever the mechanism in the case of LIMPs, our finding that a small number of molecules in our preparation link the flank of different LIMPs (Fig. 3) indicates that recombination indeed occurs at these sites.

While this in principle could cause copy number variation among interLIMPs, by circularization and loss of the intervening sequence, we saw no correlation between the position of the main recombination events and the changes in sequence coverage along the genome (Fig 4). We therefore propose that these variations stem from another documented role of cruciforms, i.e. to serve as sites for initiation of DNA replication [41]. Such a role has been described for the rolling-circle replication of single-stranded viruses and plasmids [42–44], but also for eukaryotic nuclear DNA, where a protein of the 14-3-3 family stabilizes the cruciform at the replication origin [45]. Replication mechanisms in algal mitochondria have thus far received very little attention, and little can be inferred from comparison with other systems. In animal mitochondria, the heavy and light strands are replicated from distinct origins [46]. In land plants, mitochondrial DNA replication is probably very different because the DNA mostly occurs as linear branched molecules [47]. In Cryptophyte algae, it has been postulated that a palindrome found in a repetitive region is part of the replication origin [48], but no experimental evidence was provided. For *L. incisa* as well, we are tempted to speculate that DNA cruciforms formed at the LIMPs by negative supercoiling serve as origins of replication. This would explain, if these origins fire at different rates, the unequal representation of interLIMP regions in the population of DNA molecules sequenced. If replication is unidirectional as at animal mitochondrial origins, this direction could be determined by the orientation of the loop. During rolling circle replication, the unpaired displaced strand is expected to fold as a hairpin at the downstream LIMPs, which then could serve as a replication origin for the complementary strand.

Transcription is a source of local supercoiling *in vivo*, hence might contribute to the formation of the DNA cruciform. This could provide a link between transcriptional activity and DNA replication in the *L. incisa* mitochondrion. But when the LIMPs themselves are transcribed, the longest of them will form RNA stem-loops of decent stability (Table 2). These may be targets for post-transcriptional cleavage, which could explain why RNA-Seq coverage is interrupted. In *Prototheca*, processing sites on the two polycistronic pre-mRNAs have been mapped to the loop of short palindromic sequences [16]. Palindromic repeats have also been proposed to direct processing in *Chlamydomonas* mitochondria [27], as have the Repetitive Extragenic Palindromes (REP) elements found in many bacterial genomes [49]. RNase III-dependent RNA processing has been observed at the 26-27-nt long stem-loop structures formed by repetitive palindromic *nemis* elements in *Neisseria meningitidis* [50]. In the case of *L. incisa*, the fact that LIMPs with stems shorter than 21 nt seem to have little or no effect on RNA-Seq coverage, while longer ones efficiently interrupt it (Table 2), is compatible with an implication of RNase III: the dimeric bacterial enzyme is known to cleave only stems with more than two helical turns, i.e. longer than 20 nt [50, 51]. All 5 ribonuclease III homologs encoded in the nuclear genome of *L. incisa* contain N-terminal extensions compared to their bacterial homologs and are predicted to be directed to organelles (data not shown). Other endoribonucleases that are known to act at defined stem-loop or secondary structures [52, 53] could be involved as well in the processing of the precursor RNAs, as well as in their degradation. Incidentally, the fact that we observed unambiguous evidence for the addition of a 3’ poly-A tail at a few specific locations (including rRNAs and intergenic regions, data not shown) suggests that degradation of the *L. incisa* mitochondrial RNAs uses a system based on Poly(A)-Polymerase or PolyNucleotide Phosphorylase. Poly-A tailing, known to occur in chloroplast and mitochondria [54], has been proposed to operate in mitochondrial RNA degradation, in particular in *Chlamydomonas* [55].

With minor variations, most of the discussion above can also be applied to the HyLIMP. For example, only the HyLIMP with the highest stability is correlated with a drop in RNA-Seq coverage. The HyLIMP repeat is less well-defined and far less abundant than the LIMP, yet it shares with it a palindromic nature and internal repetitivity (at least for the longer of the HyLIMPs). In fact, the presence of the AACCA pentanucleotide in both is a strong indication for a partially common origin, as is the fact that HyLIMP #3 shows the same loop sequence as the LIMPs. The existence of many independent copies of the non-repetitive core of the HyLIMP (common to HyLIMPs #1, 2 and 4, see Fig. 5) in the *L. incisa* mitochondrial genome suggests that it preexisted and recruited the repeat units to extend its stem. Indeed, this core sequence is also present in the chloroplast genomes of *L. incisa* and two other clade C Trebouxiophyceae. Obviously, this central short palindromic repeat spread from the chloroplast to the mitochondrial genome, or vice-versa: in the absence of sequence for mitochondrial genomes of basal Clade C Trebouxiophyceae, it is not possible to ascertain the direction of the transfer. In the mitochondrion of *L. incisa*, HyLIMPs probably originated when the repeat co-opted for its elongation the repetitive unit of the LIMP along with another pentanucleotide. Remarkably, this recruitment respected the orientation found in the LIMP (A-units first). Whatever the phylogenetic path, it is probably not by chance that this core HyLIMP stem happens to be 10 nt long, and that it was extended by incorporating a 10-nt repeat. The pitch of a double-stranded RNA or DNA helix is about 10 nt, so that the repetitive part of LIMPs and HyLIMPs is predicted to show identical residues along a given generator of the helix. This property might be used by these repeats to perform their elusive molecular function, be it related to DNA or to RNA metabolism.

## Conclusion

The mitochondrial genome of *L. incisa* encodes a unique type of repetitive palindromic DNA repeat sequence, the LIMP, and a related repeat, the HyLIMP. RNA sequencing suggests that the longest LIMPs and HyLIMPs are sites of transcript processing. Experimental studies are needed to confirm the functional role(s) of the LIMP and characterize the molecular mechanisms, and to identify the protein(s) that might interact with the LIMP.

## Methods

### Algal strain

The strain studied was an original isolate of *L. incisa* obtained from snow water patches in the alpine environment of Mt Tateyama, Japan [3] and deposited in the Göttingen University culture collection as SAG 2468. Algae were grown in modified BG11 medium at 25 °C and a light intensity of 130 μmol photons m^−2^ · s^−1^.

### Genome sequencing and assembly

DNA was extracted following the CTAB DNA extraction protocol [56] with modifications. Whole-genome shotgun sequencing was performed using the Illumina short-read technology (HiSeq 2000 instrument) with PE (2 ×100 nt, insert size ~300 bp, performed by Tufts University core facility) and long jumping distance MP (2 × 100 nt, insert size ~8 kb, performed by MWG Eurofins) libraries. Reads were adapter- and quality-trimmed using cutadapt 1.1 (http://code.google.com/p/cutadapt/) and prinseq-lite 0.15 [57] with the following thresholds: min. read length 30 nt; min. base quality 20, min. average read quality 28. After preprocessing, a total of 189,672,359 reads remained for assembly. Several genome assemblies were computed using the de Bruijn graph-based assemblers SOAPdenovo 1.05 [58], Velvet 1.2.08 [59], and CLC Genomics Workbench 5.1.64 (CLC bio, Denmark; http://www.clcbio.com/) with k-mer values of 23, 35, 51, or 63 for each assembler. An assembly was also made with the super-read-based assembler MSR-CA (MaSuRCA) 1.9.3 [60] with an automatically set k-mer value of 31. In order to assemble specifically the mitochondrial genome, potentially mitochondrial sequences were identified by searching for similarity between the scaffolds and contigs of all assemblies and the complete sequences of 41 mitochondrial genomes from Chlorophyta available at the NCBI GenBank database. Searches were done both at the nucleotide level using BLASTN 2.2.25 [61] and the aminoacid level using BLASTX. A total of 1828 scaffolds/contigs were retained. All reads mapping to these selected scaffolds/contigs using SOAP 2.21 [62] were extracted, along with their mates, and then assembled using CLC with k-mer values of 23, 35, 51, or 63. Thirty-nine scaffolds/contigs generated in these four assemblies that were of length ≥100 nt and with a read coverage >1500, or that were of length ≥500 nt, were selected, after eliminating those coming from the *L. incisa* chloroplast genome [8]. A superassembly was then computed using the overlap-layout-consensus assembler in Geneious 5.5.2 (Biomatters Ltd., New Zealand; http://www.geneious.com/). To resolve the genome sequence, the superassembly and the original 39 scaffolds/contigs were compared using BLAT 35x1 [63] and manually joined based on the pattern of overlap, until a single continuous sequence could be reconstructed. This sequence was iteratively refined by examining the mapping of genomic read pairs using BWA 0.6.2 [64] (options “-n 0.04 -o 0.02 -l 20”). In all analyses described above, alignment files in SAM and BAM format were sorted and converted using utilities from the samtools 0.1.18 [65] and bamtools 2.1.0 [66] packages.

Protein-coding genes were predicted using GeneMarkS 4.28 [67], rRNA genes were identified with RNAmmer 1.2 [68] and tRNA genes were predicted using tRNAscan-SE 1.21 [69]. RNAweasel [70] was used to find group I and group II introns, and repeated DNA sequences were identified using RepeatScout 1.0.5 [71] and RepeatMasker 3.3.0 [72]. The putative function of protein-coding genes was assigned based on aminoacid sequence similarity with homologs from proteins encoded by complete mitochondrial genomes of Chlorophyta using BLASTP searches. All annotations were manually reviewed. Open reading frames (ORFs) with no similarity to any sequence in NCBI GenBank were removed. The annotated sequence of the *L. incisa* mitochondrial genome has been deposited in the GenBank database under accession number [GenBank:KP902678] (BioProject PRJNA283614 ).

### Transcriptome sequencing and analysis

The transcriptome of *L. incisa* was analyzed by high-throughput sequencing of cDNAs (RNA-Seq method) using the Illumina technology (HiSeq 1000 instrument). cDNAs were generated from mRNAs isolated under four growth conditions: exponential growth, 72 h nitrogen starvation under normal light (75 μmol photons m^−2^ · .s^−1^), as well as 12 h and 72 h nitrogen starvation under high light (150 μmol photons m^−2^ · .s^−1^). RNA was isolated from frozen samples using SV Total RNA isolation kit (Promega) after breaking the material with iron beads in liquid nitrogen. RNA quality was examined on a 2100 Electrophoresis Bioanalyzer. Illumina Truseq sequencing (50-nt single-end and 100-nt paired-end reads, insert size ~150 bp) was performed by the transcriptomics platform of the Institut de Biologie de l’École Normale Supérieure. For the purpose of this study, reads from all growth conditions were pooled, adapter-trimmed (using cutadapt 1.1), and mapped onto the mitochondrial genome sequence using GSNAP 2011-12-28 [73] (option “--max-mismatches = 0.04”). In total, 716,251 reads mapped to the mitochondrial assembly, of which 604,629 mapped to the rRNA genes.
